# *Tubaramure*, a Food-Assisted Maternal and Child Health and Nutrition Program in Burundi, Increased Household Food Security and Energy and Micronutrient Consumption, and Maternal and Child Dietary Diversity: A Cluster-Randomized Controlled Trial

**DOI:** 10.1093/jn/nxz295

**Published:** 2019-12-20

**Authors:** Jef L Leroy, Deanna K Olney, Lilia Bliznashka, Marie Ruel

**Affiliations:** 1 International Food Policy Research Institute, 2033 K Street NW, Washington DC 20006, USA; 2 Department of Global Health and Population, Harvard T. H. Chan School of Public Health, Boston MA 02115, USA

**Keywords:** children, mothers, household, food consumption, dietary diversity, infant and young child feeding practices

## Abstract

**Background:**

Food-assisted maternal and child health and nutrition programs are a widely used approach to address undernutrition. Little is known about the effects of these programs’ combined household and individual food rations on household and individual food consumption. *Tubaramure* in Burundi targeted women and children during the first 1000 d of life, and included: *1*) food rations (corn-soy blend and micronutrient-fortified vegetable oil); *2*) health services strengthening and promotion of their use; and *3*) behavior change communication on nutrition, hygiene, and health practices.

**Objectives:**

The objectives were: *1*) to assess *Tubaramure*’s impact on household food consumption and food security, maternal dietary diversity, and infant and young child feeding practices; *2*) to explore the role of the food rations; and *3*) assess 6–8 mo impacts around 8 mo after the end of the program.

**Methods:**

We used a 4-arm cluster-randomized controlled repeated cross-sectional design (11,906 observations). The treatment arms received the same food ration but differed in the ration timing and duration: *1*) the first 1000 d; *2*) from pregnancy through 17.9 mo of age; or *3*) from birth through 23.9 mo of age.

**Results:**

*Tubaramure* significantly (*P* < 0.05) improved the percentage of food secure households [from 4.5 to 7.3 percentage points (pp)], and increased household energy consumption (from 17% to 20%) and micronutrient consumption. The program had a positive effect on maternal dietary diversity (+0.4 food groups, *P* < 0.05) and increased the proportion of children aged 6–23.9 mo consuming ≥4 food groups (from 8.0 to 9.6 pp, *P* < 0.05). The effects on many outcomes were attributable to the food rations. Postprogram effects (*P* < 0.05) were found on household food security, maternal dietary diversity, and younger sibling's complementary feeding practices.

**Conclusions:**

Programs such as *Tubaramure* have the potential to improve food security and household and individual energy and micronutrient consumption in severely resource-constrained populations, as seen in rural Burundi. This trial was registered at clinicaltrials.gov as NCT01072279.

## Introduction

Food-assisted maternal and child health and nutrition (FA-MCHN) programs are a widely used strategy in low- and middle-income countries to address problems of hunger, food insecurity, and undernutrition (1–3). These programs increasingly target mothers and children during the first 1000 d of life (from pregnancy through to the child's second birthday) (4). FA-MCHN programs such as those implemented by the U.S. Agency for International Development (USAID)’s Office of Food for Peace (FFP) generally include the distribution of micronutrient-fortified food commodities typically composed of a household ration and an individual ration specifically targeted to mothers and children. In addition, these types of programs commonly provide interventions such as health service-strengthening activities, the promotion of the use of these services, and behavior change communication (BCC) aimed at promoting optimal nutrition, hygiene, and health practices.

Programs providing household food rations and those aimed at improving child complementary feeding have been shown to have positive effect across a range of outcomes. The former have been shown to have a positive effect on household food consumption and child nutritional status (1, 5, 6), while the latter have been effective in improving child linear growth (7), especially when they include BCC (8). Evidence is scant, however, on the impact of programs combining a household and individual food ration with BCC and health interventions on household food consumption, and maternal and child diets.

This paper presents findings from a large, cluster-randomized controlled effectiveness trial that assessed the impact of a FA-MCHN program (*Tubaramure*) in Burundi. The main objective of *Tubaramure* was to prevent undernutrition in women during pregnancy and the first 6 months of lactation, and in children aged 0–23.9 mo. The program had 3 core components: the distribution of food rations; a BCC strategy focused on adequate nutrition, health, and hygiene practices; and activities to improve provision and use of health services. Previously, we showed that the program had a beneficial impact on the primary study outcomes: it had a significant, protective effect on the prevalence of child stunting and anemia in mothers and children (9, 10).

In this paper, we examined the program's impact on household food consumption, food security, hunger, and dietary diversity, and on maternal dietary diversity and infant and young child feeding (IYCF) practices. Where possible, we assessed to what extent these effects were a result of the *Tubaramure* food rations. Lastly, we evaluated whether impacts were observable several months (a mean of 8 mo across the sample) after the program had ended.

## Methods

### Study population and program

Burundi ranks 184 (out of 188) on the Human Development Index and is 1 of the poorest countries in the world. Its population suffers from extreme hunger (11, 12). *Tubaramure* was implemented in eastern Burundi in the provinces of Cankuzo and Ruyigi from 2010 to 2014 by a Catholic Relief Services-led consortium of nongovernmental organizations (Food for the Hungry, International Medical Corps, and Caritas Burundi) and funded by USAID's FFP. The program was targeted to women and children in the first 1000 d. Enrollment into the program was open to pregnant women (≥4 months of gestation) and mothers of children aged <6 mo with participation up to age 24 mo. Details on the program have been described elsewhere (11) and are provided in the **Supplemental Text**.

The program included 3 core components. The first component, the provision of food rations was expected to increase access to and consumption of energy-rich and micronutrient-rich foods. *Tubaramure* households received a monthly food ration of 12 kg of micronutrient-fortified corn-soy blend (CSB) and 1200 g of vitamin A-enriched oil (composition provided in **Supplemental Tables 1**and **2**). In addition to this ration intended for household consumption, beneficiary mothers received an individual ration (6 kg of CSB and 600 g of oil) during pregnancy and until their children were aged 6 mo. When the child reached age 6 mo, the age at which introduction of complementary foods is recommended, the individual ration of CSB and oil was halved and shifted to the child. Program beneficiaries continued to receive the family and individual rations until the child was aged 24 mo. As part of the evaluation design, the timing and duration of receiving the household and individual food rations differed across treatment arms (see next section). The second component was aimed at improving the provision of health services (which included training health staff and providing supplies such as equipment for prenatal care, labor and delivery, growth monitoring, and curative care) and active promotion of the use of these services. The third component was a BCC strategy to improve health and hygiene practices, maternal nutrition, and IYCF practices. Leader mothers (program beneficiaries elected by their peers) were trained by *Tubaramure* health promotors and provided BCC through group sessions to beneficiary mothers twice a month. Other BCC components included cooking demonstrations and home visits by leader mothers. Because of delays in the roll-out of the module on complementary feeding in all intervention arms, a large proportion of beneficiaries was not exposed to these important messages (13).

### Evaluation design and sampling

The impact of *Tubaramure* was evaluated with a cluster-randomized controlled study design with repeated cross-sectional surveys. A cluster was defined as a *colline* (or hill), an official administrative unit in Burundi. A cluster design was used because individual randomization of the treatment was not feasible. A total of 60 *collines* was randomly assigned to 1 of 4 study arms (15 *collines* per arm) in a public lottery event in Ruyigi. The T24 arm received all program benefits during pregnancy and up to the age of 23.9 mo for the child. The same benefits were received in the T18 arm, but food rations were discontinued when the child was aged 17.9 mo. The TNFP (“no food during pregnancy”) arm received the same benefits as the T24 arm, but started receiving food rations only at birth. Households in *collines* assigned to the fourth arm (control) did not receive any program benefits but had access to the improved health services.

Three repeated cross-sectional surveys were conducted to assess program impacts (**Table 1**). The baseline survey was conducted before the program started in 2010 and drew a sample of households with a child aged 0–23.9 or 24–41.9 mo. In *collines* assigned to the treatment arms, program services were available shortly after the baseline survey was completed and eligible women and children were invited to enroll in the program. The first follow-up, in 2012, was conducted when the program was ongoing and sampled households with a child aged 0–23.9 mo. The primary focus of this survey was to assess outcomes best measured in children eligible to receive program benefits, such as program participation and health and nutrition practices. The second follow-up survey targeted households with a child aged 24–41.9 mo and was conducted after the program had ended in 2014. The largest impact on child linear growth was expected in children this age, because they were exposed to the program from early pregnancy to age 23.9 mo. If a younger sibling (aged 0–23.9 mo) was available in the household at the time of the second follow-up, IYCF practices were assessed for this sibling.

**TABLE 1:** Survey waves and reported outcomes^1^

Type of household	Baseline (2010)	Follow-up (2012)	Follow-up (2014)
Index child (0–23.9 mo) “younger child”	Household• Hunger• Dietary diversity	Household• Hunger• Dietary diversity• Food insecurity• Food consumption	
		MotherDietary diversity	
	Index childIYCF	Index childIYCF	
Index child (24–41.9 mo) “older child”	Household• Hunger• Dietary diversity		Household• Hunger• Dietary diversity• Food insecurity
			MotherDietary diversity
			Index child—
			Sibling of index child (0–23.9 mo)IYCF

1 IYCF, infant and young child feeding.

The analyses presented here used the 2010 and 2012 data to assess impact while the program was ongoing. We also used the 2014 data to assess postprogram impact. At the time of the 2014 survey, children aged 24–41.9 mo had graduated from the program 0–18 mo before the survey. The siblings included in this survey were never program beneficiaries.

Before each survey a household census was conducted in the research *collines* to generate a complete list of households with an eligible child. The target sample size for each *colline* was calculated with a probability proportional to size approach and a list of randomly ordered households to be surveyed was generated for each *colline*. We estimated impacts based on intent-to-treat and therefore inclusion in the survey was based on program eligibility rather than participation. If there was only 1 child in the target age range in a household, that child was selected as the “index child.” If there was more than 1 child in a household, a child was randomly chosen by alphabetic order of their first names.

The study's sample size was based on power calculations to detect meaningful changes in anemia and child linear growth (9, 10). With use of the same type 1 and 2 errors (α = 0.05 and β = 0.10, respectively) and the sample intracluster correlation, we calculated the size of the detectable effects for all outcomes presented in this study. The available sample allowed us to detect impacts in per adult equivalent (AE) household energy consumption of 649 kcal, in household food insecurity access score (HFIAS) (14) of 2.41 points, in household hunger score (HHS) (15) of 0.55 points, in mother and child dietary diversity of 0.42 and 0.27 food groups, respectively, and in the proportion of children being fed a diet with minimum dietary diversity, being fed with minimum frequency, being fed a minimum acceptable diet, and a diet with Fe-rich foods of 15, 14, 10, and 8 percentage points (pp), respectively (16). Because all the estimated impact models controlled for covariates, the actual minimum detectable differences were smaller.

### Data collection and analyses

Data collection surveys were conducted during the same season to reduce the possible effect of seasonality. The baseline data were collected between October and December 2010; the follow-up surveys took place from October to January 2012 and from October to December 2014. Household questionnaires were administered in the Kirundi language by extensively trained and closely supervised fieldworkers. At each survey, information was collected on household demographic and socioeconomic characteristics. An overview of the outcomes assessed in each survey is provided in Table 1. Written informed consent for participation was obtained from the primary household respondent before the start of each interview. The study was approved by the International Food Policy Research Institute's IRB and the Ministry of Health of Burundi. This trial was registered at clinicaltrials.gov as NCT01072279.

For HHS and HFIAS, we report the continuous score (ranging from 0 to 9 and from 0 to 27 for the HHS and HFIAS, respectively) and the categorical indicators (14, ). For household dietary diversity score, we report the continuous score (range 0–12) (17). Household energy and nutrient consumption were calculated from data on the consumption of 108 foods in the past 7 d. Quantities were converted from local units to kilograms, with missing units imputed from commodity-specific unit modes at different aggregation levels (i.e., local administrative units: sous-*colline, colline*, commune, and province). Net quantities were obtained by multiplying each reported quantity by its food-specific edible fraction (18–22). Energy and nutrient consumption were then calculated with food composition data from the HarvestPlus Food Composition Table for Central and Eastern Uganda (20), and, where necessary, supplemented with data from other food composition tables (18, 19, 23, 24). Because no data were collected on how meals were prepared, nutrient composition was not adjusted for retention after preparation. To account for differences in household size and composition, all consumption variables were calculated per AE. AE was calculated by dividing each individual's age- and sex-specific recommended daily energy intake by the mean recommended intake of a 30–60-y-old male, 65 kg in weight with a moderate amount of physical activity (3000 kcal or 12,552 kJ/d). Adjustments for lactation were applied where this information was available (25). The number of AE for each household was computed as the sum of the individual AEs. In addition to per AE total household energy consumption, we examined energy consumption from 12 food groups: cereals and grains; roots and tubers; legumes, nuts, and pulses; fruits; vegetables; meat and poultry; fish and seafood; milk and dairy products; eggs; oils and fats; sweets, spices, and beverages; and miscellaneous (17).

Maternal dietary diversity was assessed with a modified version of the dietary diversity component of the WHO IYCF questionnaire (26), classifying consumption in 1 of 9 food groups: starchy staples; dark green leafy vegetables; vitamin A-rich fruits and vegetables; other fruits and vegetables; organ meat; meat and fish; eggs; legumes, nuts, and seeds; and milk and dairy products, and a total dietary diversity score was calculated (27).

Complementary feeding practices for children aged 6–23.9 mo were assessed with the WHO-recommended IYCF practices measurement tool (26). The following indicators were calculated: consumption of foods from 7 predefined food groups (grains, roots, and tubers; legumes and nuts; dairy products; flesh foods; eggs; vitamin A-rich fruits and vegetables; and other fruits and vegetables), dietary diversity score (range from 0 to 7), being fed a diet with minimum dietary diversity (i.e., ≥4 food groups), minimum meal frequency (≥2 meals for breastfed children aged 6–8.9 mo; 3 meals for breastfed children aged 9–23.9 mo; 4 meals for nonbreastfed children), minimum acceptable diet (meeting both minimum dietary diversity and minimum meal frequency, in addition to having received ≥2 milk feedings for nonbreastfed children), and the consumption of iron-rich or iron-fortified foods. All indicators pertain to the child's diet in the previous 24 h.

No quantitative dietary intake data were collected in this study. To assess the extent to which the program could have improved the dietary adequacy of mothers and children <23.9 mo with respect to some key nutrients, we present several scenarios re-estimating program impact after making different assumptions about the intrahousehold distribution of changes in household consumption. We first assumed that the beneficiary mother and child got a share proportional to their AE (i.e., from their energy requirement). We then estimated the program's impact if their share was equivalent to half of their AE (i.e., they would get less than what would be “fair”) and 1.5 times their AE (i.e., they would get more than their “fair” share). The results of the different scenarios were compared to micronutrient requirements for mothers and children aged <24 mo (28–30).

### Statistical analysis

In line with the CONSORT 2010 guidelines, we did not conduct a statistical comparison of baseline means between study arms (31). Program impact for outcomes with data available at both baseline and follow-up (e.g., household dietary diversity and IYCF practices) was estimated with a double-difference *colline*-fixed effects model:

(1) $$\begin{equation*}
{y_{t{\rm{\ }} = {\rm{\ }}0,1}} = {\beta _0}{\rm{\ }} + {\beta _1}{T_t} + {\beta _2}S + {\rm{\ }}{\beta _3}{T_t}S + {\beta _4}C + {\beta _i}{X_t} + {\rm{\varepsilon }},\end{equation*}$$

where *T_t_* is time (baseline or follow-up), *S* is the study arm, *C* is a vector representing *colline*-level fixed effects, and *X_t_* is a vector of household, mother, or child-level covariates. The coefficient ${\beta _3}$ represents the estimated treatment effect of the program.

To estimate the impact of the program on outcomes measured only at follow-up (e.g., household food consumption and maternal dietary diversity), we used a single difference model:

(2) $$\begin{equation*}
{y_{t{\rm{\ }} = {\rm{\ }}1}} = {\beta _0}{\rm{\ }} + {\beta _1}S + {\beta _i}X + \varepsilon
\end{equation*}$$

where *S* and *X* are defined as above and ${\beta _1}$ represents the estimated treatment effect of the program. All models controlled for the following baseline covariates: household head's education and occupation, maternal education, household size, and house ownership. The estimates for maternal dietary diversity and children's IYCF practices were also adjusted for child age and sex, and maternal age.

We assessed the role of the program-distributed rations as follows. The impact on household food consumption was assessed with and without the consumption of the program-distributed CSB and micronutrient-fortified oil. Maternal dietary diversity, child dietary diversity, child consumption of iron-rich foods, and child minimum acceptable diet were estimated with and without the contribution of CSB (which contributed to 2 food groups: cereals and pulses).

**FIGURE 1:**
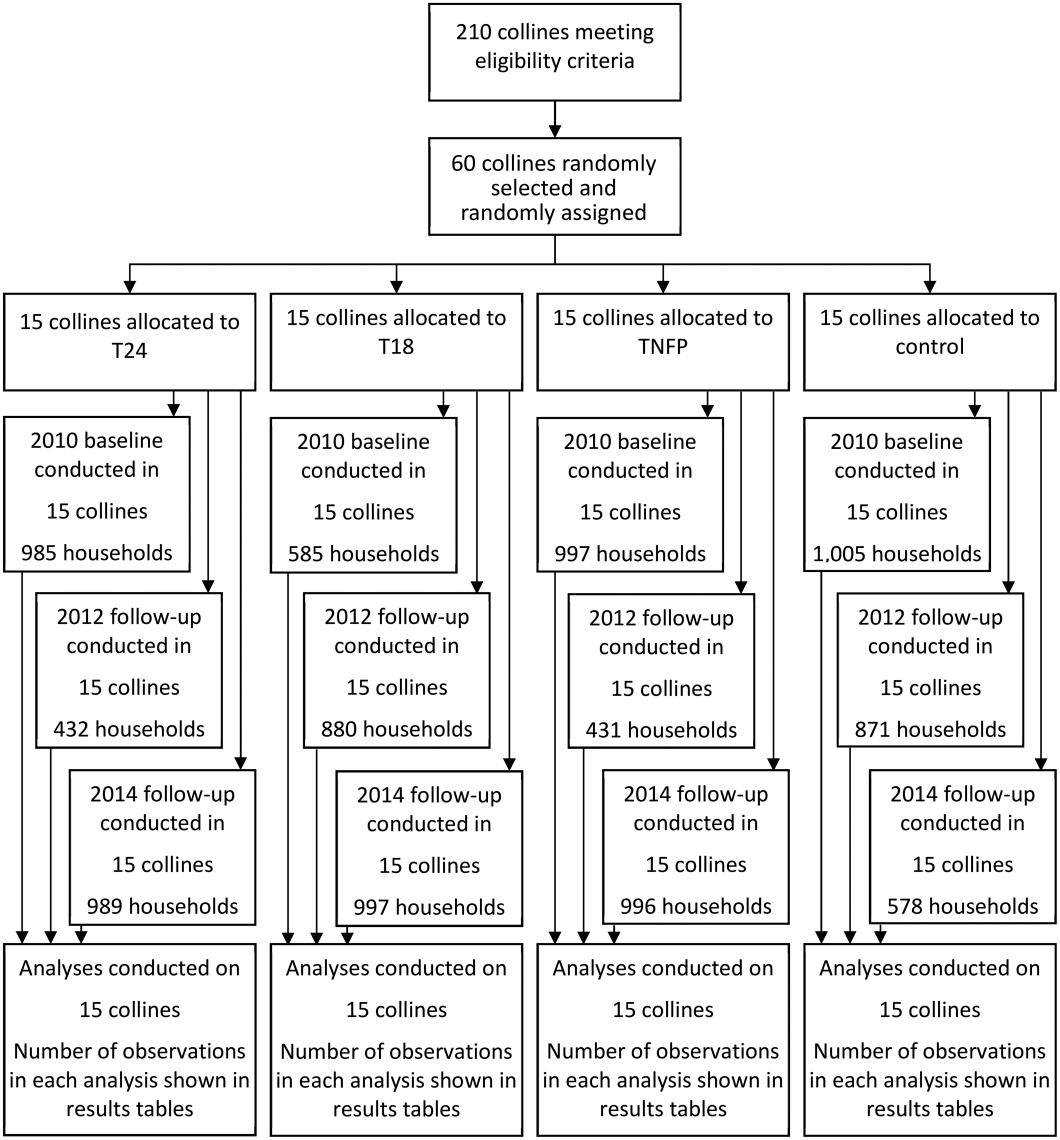
Trial flow chart.

When there was a clear a priori hypothesis of the direction of the program effect, we used 1-sided tests in line with statistical theory (32). Impact estimates were considered statistically significant at a probability of *P* < 0.05. All statistical analyses conducted were intent-to-treat. The standard errors in all analyses were adjusted for clustering at the *colline* level. Sample sizes at each survey are shown in **Figure 1**. At baseline, the analytic sample was restricted to households with complete information on all household outcomes and covariates of interest, thus excluding 23 households because of missing information. At the 2012 follow-up, the sample was restricted to the households within sample percentiles 2.5 and 97.5 of daily total per AE household energy consumption to minimize the effects of outliers (132 observations excluded). An additional 120 observations were excluded because of missing values. At the 2014 follow-up, 191 observations were excluded because of missing data. The final analysis sample thus consisted of a total of 4960 households for the analyses through the baseline and 2012 follow-up data (97% of the households interviewed) and of a total of 6946 households for the analyses through the baseline and 2014 follow-up data (99% of all households interviewed). Sample sizes by study arm are shown in **Table 2**.

**TABLE 2:** Characteristics of household, mother, and child participating in the *Tubaramure* evaluation^1^

	Study arm				Study arm			
	T24^2^	T18^2^	TNFP^2^	Control	T24^2^	T18^2^	TNFP^2^	Control
Households with child 0–23.9 mo	Baseline (2010)				Follow-up (2012)			
*N*	431	859	430	878	389	804	391	778
Household								
Size	5.5 ± 2.0	5.6 ± 2.0	5.6 ± 2.1	5.5 ± 2.0	5.8 ± 1.9	5.8 ± 2.0	5.8 ± 2.1	5.8 ± 2.0
Adult equivalents	3.6 ± 1.4	3.7 ± 1.5	3.7 ± 1.5	3.6 ± 1.5	3.7 ± 1.5	3.7 ± 1.5	3.8 ± 1.5	3.7 ± 1.4
Members under 18 y	3.3 ± 1.8	3.5 ± 1.8	3.5 ± 1.9	3.4 ± 1.9	3.6 ± 1.7	3.6 ± 1.9	3.6 ± 1.9	3.7 ± 1.8
Current *Tubaramure* beneficiary					74.2	55.3	79.8	2.7
Past *Tubaramure* beneficiary					6.4	20.4	4.7	1.3
Head								
Age, y	35.4 ± 11.1	35.2 ± 10.8	34.3 ± 10.2	34.0 ± 10.2	35.1 ± 10.0	34.8 ± 10.4	35.3 ± 10.6	33.9 ± 10.1
Male, %	93.0	93.5	93.5	90.0	94.9	94.2	93.4	92.5
No formal education, %	38.5	40.5	38.1	42.0	33.9	34.7	38.4	41.9
Mother								
Age, y	28.3 ± 6.8	29.0 ± 7.1	28.6 ± 6.7	28.3 ± 7.0	28.5 ± 6.2	28.5 ± 6.4	29.2 ± 6.9	28.1 ± 6.5
No formal education, %	50.8	49.5	54.2	54.1	43.4	40.5	48.1	51.7
Index child								
Age, mo	12.3 ± 7.0	12.9 ± 6.7	12.4 ± 6.6	13.2 ± 6.8	12.1 ± 6.4	12.1 ± 6.8	11.7 ± 6.6	13.0 ± 6.5
Boy, %	46.9	47.7	48.6	47.7	49.1	52.0	49.6	50.3
Households with child 24–41.9 mo	Baseline (2010)				Follow-up (2012)			
*N*	979	580	980	988	949	961	958	551
Household								
Size	5.9 ± 2.0	5.9 ± 2.0	5.9 ± 1.9	5.9 ± 2.0	6.0 ± 1.9	6.0 ± 2.0	6.0 ± 2.0	5.9 ± 2.0
Adult equivalents	3.9 ± 1.5	4.0 ± 1.6	3.9 ± 1.5	3.9 ± 1.5	4.1 ± 1.5	4.1 ± 1.5	4.1 ± 1.5	4.0 ± 1.5
Members under 18 y	3.7 ± 1.7	3.7 ± 1.7	3.7 ± 1.7	3.7 ± 1.8	3.8 ± 1.7	3.8 ± 1.8	3.8 ± 1.7	3.8 ± 1.8
Past *Tubaramure* beneficiary					72.4	66.8	76.5	3.8
Head								
Age, y	36.7 ± 10.7	37.1 ± 10.3	36.3 ± 10.5	36.3 ± 10.2	37.5 ± 11.0	36.0 ± 10.0	36.1 ± 10.5	36.0 ± 10.8
Male, %	92.4	90.5	92.8	92.0	91.0	92.8	92.8	92.0
No formal education, %	39.9	48.3	42.6	47.5	35.7	35.5	38.9	39.0
Mother								
Age, y	31.2 ± 7.9	31.4 ± 8.3	30.6 ± 7.3	30.5 ± 7.9	31.0 ± 7.7	29.9 ± 7.0	30.0 ± 6.9	29.4 ± 6.8
No formal education, %	48.6	55.5	56.2	55.9	45.2	42.9	48.0	51.4
Sibling of index child								
Age, mo					8.6 ± 5.7	8.2 ± 5.7	7.9 ± 5.8	7.1 ± 5.5
Boy, %					47.9	53.3	50.0	53.5

1 Values are mean ± SD or %.

2 The T24 arm received all program benefits during pregnancy and up to the age of 23.9 mo for the child. The same benefits were received in the T18 arm, but food rations were discontinued when the child was 17.9 mo of age. The TNFP (“no food during pregnancy”) arm started receiving food rations at birth, but the other benefits were the same as in T24.

## Results

### Descriptive statistics

Education levels were low (2010 baseline, Table 2), food insecurity and hunger were common (2010 baseline, **Table 3**), and around 70% of household energy consumption in the control arm came from roots, tubers, and cereals. Mothers and children consumed monotonous diets (**Table 4**). Characteristics of the study arms appeared balanced at baseline.

**TABLE 3:** Impact of *Tubaramure* on household food insecurity, hunger, dietary diversity, and daily per AE energy and micronutrient consumption^1^

	Baseline (2010)				Follow-up (2012)						
	Study arm				Study arm				Impact^2^		
	T24^3^	T18^3^	TNFP^3^	Control	T24^3^	T18^3^	TNFP^3^	Control	T24 vs. control	T18 vs. control	TNFP vs. control
*n*	431	859	430	878	389	804	391	778			
HFIAS score					10.1 ± 6.9	11.1 ± 7.3	10.7 ± 7.2	13.0 ± 7.0	−2.9 ± 0.8**	−1.9 ± 0.7**	−2.2 ± 0.9**
HFIAS scale: food secure, %					11.3	12.2	14.1	6.8	4.5 ± 2.2**	5.4 ± 2.0**	7.3 ± 2.9**
HHS score	1.3 ± 1.5	1.5 ± 1.5	1.5 ± 1.6	1.6 ± 1.6	0.6 ± 1.3	0.9 ± 1.4	0.8 ± 1.3	1.1 ± 1.5	−0.2 ± 0.2	−0.2 ± 0.2	−0.3 ± 0.2*
HHS scale: little–no hunger, %	59.6	51.2	51.2	51.3	82.3	72.9	77.2	66.2	6.9 ± 7.0	5.4 ± 4.3	10.9 ± 5.3**
Household diet diversity											
Cereals and grains, %	40.4	34.5	36.3	36.4	33.2	34.8	34.0	29.8	−0.7 ± 6.3	5.8 ± 4.5	4.1 ± 5.7
Roots and tubers, %	81.7	83.5	82.8	82.9	81.0	84.6	82.6	85.0	−2.8 ± 4.9	−0.8 ± 4.6	−2.9 ± 4.1
Legumes, nuts, and pulses, %	70.8	60.9	62.3	54.4	76.9	79.7	79.8	70.8	−9.8 ± 4.3**	3.1 ± 5.2	1.6 ± 5.0
Fruits, %	60.6	63.7	58.1	55.4	42.4	47.3	42.5	33.3	3.6 ± 6.9	5.4 ± 6.8	6.5 ± 5.7
Vegetables, %	84.0	86.0	78.6	86.3	90.0	86.3	84.4	85.6	6.5 ± 4.0	0.4 ± 3.3	6.2 ± 4.4
Meat and poultry, %	7.4	4.0	6.3	4.0	5.4	4.9	8.7	5.8	−4.4 ± 2.9	−1.4 ± 1.3	0.5 ± 1.8
Fish and seafood, %	17.6	24.6	24.9	23.7	27.8	36.1	33.2	28.3	4.8 ± 4.0	6.2 ± 2.3**	3.9 ± 4.6
Milk and dairy, %	1.9	2.4	3.0	1.5	2.8	2.2	2.3	1.8	0.6 ± 1.1	−0.8 ± 1.0	−0.9 ± 0.9
Eggs, %	2.8	1.7	3.3	1.3	1.8	2.1	2.3	1.5	−1.6 ± 1.1	−0.3 ± 1.1	−1.5 ± 1.5
Oils and fats, %	34.3	33.6	35.6	28.9	25.4	29.6	31.2	18.5	0.9 ± 5.0	5.5 ± 5.0	5.5 ± 4.5
Sugars, %	9.5	5.2	7.0	3.4	7.5	8.8	7.2	2.3	−1.7 ± 2.4	3.6 ± 1.9*	0.8 ± 1.6
Miscellaneous, %	4.4	4.1	4.9	2.1	4.1	8.0	6.1	2.6	−1.3 ± 1.7	2.7 ± 1.4*	0.3 ± 1.8
Diet diversity score	4.2 ± 1.6	4.0 ± 1.6	4.0 ± 1.9	3.8 ± 1.5	4.0 ± 1.6	4.2 ± 1.7	4.1 ± 1.6	3.7 ± 1.4	−0.1 ± 0.2	0.3 ± 0.1**	0.2 ± 0.2
Household food consumption											
Ln energy consumed, ln(kcal)					8.0 ± 0.6	8.1 ± 0.5	8.1 ± 0.5	7.9 ± 0.5	0.2 ± 0.1**	0.2 ± 0.1**	0.2 ± 0.1**
Total energy consumed, kcal					3683 ± 2104	3664 ± 1939	3624 ± 1881	3002 ± 1633	680.6 ± 325.0**	661.2 ± 192.5**	621.9 ± 194.8**
Energy from …											
cereals and grains, kcal					556.5 ± 571.5	531.0 ± 542.4	586.9 ± 598.8	326.5 ± 465.8	230.0 ± 64.0**	204.5 ± 42.3**	260.4 ± 52.4**
roots and tubers, kcal					1776 ± 1464	1831 ± 1368	1664 ± 1150	1803 ± 1302	−27.2 ± 189.2	28.0 ± 155.5	−139.3 ± 146.7
legumes, nuts, and pulses, kcal					614.4 ± 457.6	609.5 ± 494.7	626.1 ± 477.6	423.3 ± 358.0	191.1 ± 65.4**	186.2 ± 48.0**	202.8 ± 50.4**
fruits, kcal					205.0 ± 452.4	188.7 ± 300.6	191.9 ± 339.3	116.3 ± 196.4	88.7 ± 33.1**	72.4 ± 24.8**	75.7 ± 30.9**
vegetables, kcal					125.7 ± 194.5	139.2 ± 269.1	120.1 ± 212.5	111.1 ± 179.0	14.6 ± 16.4	28.0 ± 22.0	8.9 ± 20.2
meat and poultry, kcal					16.2 ± 40.4	19.3 ± 50.3	20.8 ± 57.8	18.6 ± 58.8	−2.4 ± 3.8	0.7 ± 3.0	2.2 ± 4.5
fish and seafood, kcal					13.0 ± 36.8	14.5 ± 35.6	9.6 ± 23.8	12.4 ± 30.4	0.6 ± 3.9	2.1 ± 2.0	−2.8 ± 2.1
milk and dairy, kcal					4.5 ± 26.4	5.3 ± 29.5	7.2 ± 37.0	3.8 ± 26.4	0.8 ± 1.7	1.5 ± 1.6	3.4 ± 2.3
eggs, kcal					1.4 ± 14.8	0.6 ± 3.3	0.6 ± 2.5	0.3 ± 1.8	1.1 ± 0.8	0.3 ± 0.2	0.2 ± 0.2
oils and fats, kcal					302.5 ± 308.4	265.3 ± 341.4	331.0 ± 485.6	149.6 ± 310.0	152.9 ± 32.3**	115.8 ± 19.6**	181.5 ± 36.6**
sugars, kcal					19.5 ± 81.3	15.4 ± 55.5	17.4 ± 73.0	6.0 ± 28.8	13.5 ± 5.6**	9.5 ± 3.9**	11.4 ± 4.8**
miscellaneous, kcal					48.5 ± 74.4	43.6 ± 82.0	48.9 ± 79.3	31.4 ± 67.1	17.1 ± 7.2**	12.2 ± 5.3**	17.5 ± 6.4**
Micronutrients^4^											
Vitamin C, mg					249 ± 153	259 ± 146	261 ± 147	207 ± 125	42.1 ± 15.1**	52.0 ± 17.7**	53.8 ± 16.7**
Iron, mg					39.1 ± 28.3	35.5 ± 23.2	36.9 ± 23.8	21.7 ± 12.9	17.4 ± 3.1**	13.8 ± 1.5**	15.2 ± 1.9**
Vitamin A, μg					2170 ± 1850	1750 ± 1560	1900 ± 1660	1140 ± 1530	1030 ± 217**	616 ± 147**	764 ± 215**
Zinc, mg					15.5 ± 9.7	14.3 ± 8.3	14.8 ± 8.4	9.9 ± 5.3	5.6 ± 1.2**	4.6 ± 0.6**	4.9 ± 0.7**
Thiamin, mg					3.1 ± 1.6	3.0 ± 1.6	3.0 ± 1.6	2.3 ± 1.2	0.8 ± 0.2**	0.7 ± 0.1**	0.7 ± 0.1**
Riboflavin, mg					2.2 ± 1.3	2.1 ± 1.2	2.1 ± 1.2	1.6 ± 0.9	0.7 ± 0.2**	0.5 ± 0.1**	0.6 ± 0.1**
Vitamin B-6, mg					4.8 ± 3.0	4.6 ± 2.5	4.7 ± 2.7	3.7 ± 2.2	1.1 ± 0.3**	0.9 ± 0.2**	1.0 ± 0.2**
Folate, μg					1310 ± 766	1260 ± 723	1290 ± 713	894 ± 505	413 ± 116**	369 ± 73.2**	394 ± 74.4**
Vitamin B-12, μg					1.4 ± 1.6	1.1 ± 1.3	1.2 ± 1.4	0.4 ± 0.9	0.9 ± 0.2*	0.7 ± 0.1*	0.8 ± 0.1*

1 Values are mean ± SD or %. All kcal estimates are per AE. AE, adult equivalent; HFIAS, household food insecurity access score; HHS, household hunger score.

2 Values are double difference impact estimates ± SE for outcomes with baseline and follow-up data, and single difference impact estimates ± SE for outcomes with follow-up data only. All estimates controlled for clustering, household head's education and occupation, maternal education, household size, and housing ownership. One-sided tests were conducted when there was an a priori hypothesis about the direction of program effect.

3 The T24 arm received all program benefits during pregnancy and up to the age of 23.9 mo for the child. The same benefits were received in the T18 arm, but food rations were discontinued when the child was 17.9 mo of age. The TNFP (“no food during pregnancy”) arm started receiving food rations at birth, but the other benefits were the same as in T24.

4 Values represent micronutrient availability in raw foods, unadjusted for micronutrient retention after preparation.

* Impact estimate significantly different from 0, *P* < 0.10.

** Impact estimate significantly different from 0, *P* < 0.05.

**TABLE 4:** Impact of *Tubaramure* on maternal dietary diversity^1^

	Follow-up (2012)						
	Study arm				Impact^2^		
	T24^3^	T18^3^	TNFP^3^	Control	T24 vs. control	T18 vs. control	TNFP vs. control
*N*	389	804	391	778	2362	2362	2362
Dietary diversity score (includes CSB)	4.2 ± 1.1	4.3 ± 1.2	4.2 ± 1.2	3.9 ± 1.1	0.3 ± 0.1**	0.4 ± 0.1**	0.4 ± 0.1**
Dietary diversity score (excludes CSB)	3.9 ± 1.2	4.2 ± 1.2	4.1 ± 1.2	3.9 ± 1.1	0.1 ± 0.1	0.3 ± 0.1**	0.2 ± 0.1**
% of mothers who consumed (past 24 h)							
Starchy staples (includes CSB)	86.1	88.3	89.0	86.6	−0.5 ± 3.7	1.7 ± 1.8	2.4 ± 2.4
Starchy staples (excludes CSB)	77.9	84.5	83.1	86.4	−8.5 ± 3.8**	−1.9 ± 2.1	−3.3 ± 3.3
Legumes, nuts, and seeds (includes CSB)	89.7	86.3	86.2	71.5	18.3 ± 3.8**	14.9 ± 3.5**	14.7 ± 4.0**
Legumes, nuts, and seeds (excludes CSB)	75.3	79.9	77.7	70.6	4.8 ± 5.1	9.3 ± 4.2**	7.2 ± 5.0
Dark green leafy vegetables	87.9	87.2	82.6	85.9	2.1 ± 2.7	1.3 ± 2.8	−3.3 ± 3.5
Other vitamin A-rich fruits and vegetables	89.5	86.7	86.2	82.8	6.7 ± 3.6*	3.9 ± 3.4	3.4 ± 3.4
Other fruits and vegetables	35.7	43.4	43.5	31.5	4.2 ± 5.8	11.9 ± 6.1*	12.0 ± 4.8**
Organ meat	0.3	1.5	3.1	0.5	−0.3 ± 0.4	1.0 ± 0.6*	2.6 ± 1.4*
Meat and fish	22.4	30.6	28.4	25.7	−3.3 ± 4.0	4.9 ± 4.0	2.7 ± 5.4
Eggs	2.6	2.1	2.0	1.0	1.5 ± 0.9*	1.1 ± 0.6*	1.0 ± 0.9
Milk and dairy products	1.0	1.1	2.6	0.9	0.1 ± 0.7	0.2 ± 0.6	1.7 ± 0.8**

1 Values are mean ± SD or %. CSB, corn-soy blend.

2 Values are double difference impact estimates ± SE for outcomes with baseline and follow-up data, and single difference impact estimates ± SE for outcomes with follow-up data only. All estimates controlled for clustering, household head's education and occupation, maternal education, household size, and housing ownership. One-sided tests were conducted when there was an a priori hypothesis about the direction of program effect.

3 The T24 arm received all program benefits during pregnancy and up to the age of 23.9 mo for the child. The same benefits were received in the T18 arm, but food rations were discontinued when the child was 17.9 mo of age. The TNFP (“no food during pregnancy”) arm started receiving food rations at birth, but the other benefits were the same as in T24.

* Impact estimate significantly different from 0, *P* < 0.10.

** Impact estimate significantly different from 0, *P* < 0.05.

### Program enrollment and participation

At the 2012 follow-up, between 75% and 85% of the surveyed households in program *collines* reported that they were either currently enrolled or had previously been enrolled in the *Tubaramure* program (Table 2). In 2014, 67–72% of households in the *Tubaramure* area reported having ever been enrolled in the program. Participation in monthly food distribution among those enrolled in the program was very high: the reported number of distributions attended in the past 4 mo was close to (the expected) 4 in all of the intervention arms (results not shown). Care group attendance was considerably lower than intended by program design (2 sessions/mo); mothers reported having attended <4 sessions on average in the 4 mo preceding the 2012 survey (results not shown). We do not know if this is because of women not participating in organized sessions or sessions not being organized. Exposure to cooking demonstrations was even less frequent. Only around half of the mothers who participated in care group sessions reported that a cooking demonstration had ever been offered.

### Program impact: household

The program had a significant positive impact on household food security (Table 3): at the 2012 follow-up, the HFIAS score was significantly lower for each of the treatment arms compared to the control arm by 2–3 points (indicating improved food access security). The proportion of food secure households was between 4.5 ± 2.2 (mean ± SE) and 7.3 ± 2.9 pp higher in the treatment compared to the control arms. There was no significant impact on the HHS score and the impact on the proportion of households categorized as experiencing little or no hunger was limited to the TNFP arm (10.9 ± 5.3 pp). A small program impact on household dietary diversity (0.3 ± 0.1 groups) was only found in the T18 arm. No consistent impact was found on household consumption of specific food groups.

*Tubaramure*’s impact on household energy consumption was similar across treatment arms and ranged from 621.9 ± 194.8 to 680.6 ± 325.0 kcal/(AE/d), equivalent to a 17–20% higher energy consumption (based on the log-transformed variable) compared to the control group. Large program impacts were found on energy consumption from food groups that correspond to the food rations: energy from cereals and grains [impact of 204.5 ± 42.3 to 260.4 ± 52.4 kcal/(AE/d)], legumes [186.2 ± 48.0 to 202.8 ± 50.4 kcal/(AE/d)], and oils and fats [115.8 ± 19.6 to 181.5 ± 36.6 kcal/(AE/d)]. *Tubaramure* also had a significant impact on energy consumption from 3 food groups not related to the rations: energy from fruits [impact of 72.4 ± 24.8 to 88.7 ± 33.1 kcal/(AE/d)] and sugars [9.5 ± 3.9 to 13.5 ± 5.6 kcal/(AE/d)] and miscellaneous [12.2 ± 5.3 to 17.5 ± 6.4 kcal/(AE/d)], although the impact on the last 2 groups was negligible from a dietary perspective. No effect was found on energy from roots and tubers, vegetables, or any of the animal-source food groups. *Tubaramure* had a significant impact on the household consumption of all assessed micronutrients. When excluding the *Tubaramure* CSB and oil rations, the impact estimates for total energy consumption dropped by more than half and remained only significant in the T18 arm (**Supplemental Table 3**); the impact on energy from legumes remained significant in all treatment arms but decreased by about one-third in magnitude; and the impact on the consumption of energy from cereals and grains disappeared. The impact on household micronutrient consumption dropped considerably in size but remained statistically significant for most of the micronutrients in all treatment arms.

### Program impact: mothers

*Tubaramure* had a positive impact of approximately 0.4 food groups on maternal dietary diversity in all 3 treatment arms (Table 4), which was mostly a result of the program's effect on legume consumption (impact 14.9 ± 3.5 to 18.3 ± 3.8 pp). When CSB was excluded from the analyses, the program effect on maternal dietary diversity and the proportion of mothers consuming legumes remained significant in the T18 and TNFP arms only. The significant program effect on the consumption of non-vitamin A-rich fruits and vegetables was limited to the TNFP arm. No meaningful program effects were found for any of the other food groups. The intrahousehold redistribution scenarios showed that the program had the potential to contribute substantially to maternal micronutrient intake, even under the most conservative scenario where they received less than their “fair” share (i.e., a share proportional to their energy requirements) (**Supplemental Table 4**).

### Program impact: children aged 6–23.9 mo

*Tubaramure* had only a small effect on the mean dietary diversity score of children, which was limited to the T18 and TNFP (0.2 ± 0.1 and 0.3 ± 0.1 food groups, respectively) arms (**Table 5**). The program's impact on specific food groups was limited to grains, roots, and tubers (8.9 ± 3.7 pp in the T24 arm and 12.8 ± 3.9 pp in the TNFP arm) and to legumes and nuts (11.9 ± 4.3 pp in T18). When excluding the CSB, similar effects were found for grains, roots, and tubers; the positive effect for legumes and nuts disappeared and a negative program effect was found in the T24 arm. The program increased the proportion of children aged 6–23.9 mo who consumed ≥4 food groups (with use of the minimum dietary diversity indicator) in all treatment arms (impact 8.0 ± 4.6 to 9.6 ± 4.7 pp) and this effect was a result of consumption of CSB. There were large increases in the proportion of children receiving the minimum number of recommended meals (12.5 ± 4.2 and 25.9 ± 5.2 pp in the T18 and TNFP arms, respectively). Finally, children in the T18 and TNFP arm (but not in the T24 arm) were significantly more likely to be fed a minimum acceptable diet compared to children in the control arm (both when CSB was included or excluded from the analysis). Consumption of iron-rich foods increased by 40.4 ± 4.9 (T24), 26.2 ± 4.4 (T18), and 36.5 ± 4.5 (TNFP) pp compared to the control arm, and this effect was entirely a result of consumption of CSB. *Tubaramure* had the potential to contribute meaningfully to children's micronutrient intake, even under the intrahousehold redistribution scenario where children received substantially less than a share proportional to their energy requirements (**Supplemental Table 5**).

**TABLE 5:** Impact of *Tubaramure* on children's dietary diversity and child feeding practices^1^

	Baseline (2010)				Follow-up (2012)						
	Study arm				Study arm				Impact^2^		
	T24^3^	T18^3^	TNFP^3^	Control	T24	T18	TNFP	Control	T24 vs. control	T18 vs. control	TNFP vs. control
*n* ^4^	320	687	333	689	304	611	301	638	3875	3875	3875
Dietary diversity score (including CSB)	2.7 ± 1.2	2.6 ± 1.3	2.7 ± 1.3	2.5 ± 1.2	3.1 ± 1.2	3.2 ± 1.2	3.3 ± 1.1	2.8 ± 1.2	0.1 ± 0.1	0.2 ± 0.1**	0.3 ± 0.1**
Dietary diversity score (excluding CSB)	2.6 ± 1.2	2.6 ± 1.3	2.7 ± 1.3	2.4 ± 1.2	2.9 ± 1.3	3.0 ± 1.3	3.1 ± 1.2	2.8 ± 1.2	−0.1 ± 0.1	0.1 ± 0.1	0.1 ± 0.1
% of children who consumed (past 24 h)											
Grains, roots, and tubers (including CSB)	67.9	72.0	67.5	68.4	83.8	80.5	85.7	75.2	8.9 ± 3.7**	1.5 ± 3.8	12.8 ± 3.9**
Grains, roots, and tubers (excluding CSB)	27.9	71.7	67.3	68.4	81.5	79.5	84.4	75.0	7.0 ± 4.1*	0.9 ± 3.8	11.9 ± 3.9**
Legumes and nuts (including CSB)	55.3	43.0	50.8	36.2	84.5	82.7	86.3	63.6	1.7 ± 3.7	11.9 ± 4.3**	8.5 ± 4.4*
Legumes and nuts (excluding CSB)	53.1	41.1	49.7	34.5	65.7	70.2	71.4	62.2	−14.8 ± 4.6**	1.4 ± 5.4	−5.3 ± 4.8
Milk and dairy products	2.5	3.2	2.8	1.5	2.0	0.3	1.0	0.2	0.8 ± 1.6	−1.7 ± 1.2	−0.0 ± 0.9
Flesh foods	11.0	12.9	15.9	12.9	18.8	24.1	23.7	21.8	−1.2 ± 3.1	1.9 ± 2.8	−1.1 ± 5.4
Eggs	0.9	1.6	1.8	0.9	2.7	1.5	1.7	1.1	1.5 ± 1.1	−0.4 ± 1.0	−0.1 ± 1.4
Vitamin A-rich fruits and vegetables	85.8	88.5	87.7	88.0	86.1	88.7	88.4	88.5	−0.1 ± 3.0	−0.6 ± 2.8	−0.3 ± 3.2
Other fruits and vegetables	41.2	36.5	41.4	37.3	36.1	40.0	42.2	31.3	1.4 ± 6.1	9.9 ± 6.9	7.6 ± 5.6
Infant and young child feeding practices											
% of children with											
minimum dietary diversity (including CSB)	25.3	23.4	28.8	20.6	40.7	41.1	45.0	28.6	8.0 ± 4.6**	9.5 ± 4.9**	9.6 ± 4.7**
minimum dietary diversity (excluding CSB)	25.3	22.7	28.5	19.6	34.8	36.8	41.3	28.1	1.8 ± 4.8	5.5 ± 5.2	5.9 ± 5.3
minimum meal frequency	37.6	33.8	24.4	32.7	51.1	50.4	54.4	37.9	8.0 ± 6.1*	12.5 ± 4.2**	25.9 ± 5.2**
minimum acceptable diet (including CSB)	11.1	7.9	7.6	7.1	23.3	23.8	26.5	13.1	6.3 ± 5.2	10.3 ± 3.1**	13.7 ± 3.5**
minimum acceptable diet (excluding CSB)	11.1	7.5	7.6	6.8	19.9	21.3	24.5	13.1	2.8 ± 5.0	8.0 ± 3.2**	11.5 ± 3.5**
Fe-rich foods consumption (including CSB)	16.4	15.7	19.6	15.6	67.2	52.6	65.7	25.6	40.4 ± 4.9**	26.2 ± 4.4**	36.5 ± 4.5**
Fe-rich foods consumption (excluding CSB)	12.9	13.0	16.8	13.1	19.3	24.5	24.0	22.2	−2.8 ± 2.8	2.0 ± 2.8	−1.9 ± 5.6

1 Values are mean ± SD or %. CSB, corn-soy blend.

2 Values are double difference impact estimates ± SE for outcomes with baseline and follow-up data, and single difference impact estimates ± SE for outcomes with follow-up data only. All estimates controlled for clustering, household head's education and occupation, maternal education, household size, and housing ownership. One-sided tests were conducted when there was an a priori hypothesis about the direction of program effect.

3 The T24 arm received all program benefits during pregnancy and up to the age of 23.9 mo for the child. The same benefits were received in the T18 arm, but food rations were discontinued when the child was 17.9 mo of age. The TNFP (“no food during pregnancy”) arm started receiving food rations at birth, but the other benefits were the same as in T24.

4 Maximum sample sizes shown. Sample size varied by outcome. Smallest sample sizes were, at baseline: T24: 290, T18: 636, TNFP: 291, Control: 642; at follow-up: T24: 284, T18: 577, TNFP: 287, Control: 602; impact estimates: 3609.

* Impact estimate significantly different from 0, *P* < 0.10.

** Impact estimate significantly different from 0, *P* < 0.05.

### Postprogram impact

*Tubaramure* had a significant sustained impact on reported household food security in all intervention arms (**Supplemental Table 6**): in 2014, after the program had ended, the proportion of food secure households was 4.1–6.0 pp higher in the treatment compared to the control arms; and the HFIAS score was significantly lower in the treatment arms compared to the control arm by 2–3 points (indicating improved food access security). No postprogram effect was found on household hunger. A significant impact on household dietary diversity of 0.6 groups was found in the TNFP. No postprogram effect was found in the T24 arm.

*Tubaramure* had a positive postprogram impact on maternal dietary diversity of 0.4 ± 0.1 to 0.5 ± 0.1 food groups in all 3 treatment arms (**Supplemental Table 7**) which was largely a result of the postprogram effect on non-vitamin A-rich fruits and vegetable consumption. The program also had a large positive effect on the proportion of children consuming a minimally acceptable diet in all treatment arms of around 14 pp. The point estimates for the impact on children consuming a minimally diverse diet and having minimal meal frequency were large and positive but were only statistically significant in the T24 (dietary diversity) and TNFP arms (frequency). As for mothers, a food-group specific effect was found for fruits and vegetables in the T24 arm (17.1 ± 6.8 percentage points) and in the TNFP arm (11.9 ± 5.9 percentage points).

## Discussion

*Tubaramure*, a FA-MCHN program in Burundi, improved household food security, led to an increase in household energy consumption of up to 20%, increased the energy from fruit consumption, and had a positive effect on household micronutrient consumption. No effect was found on household energy consumption from vegetables or from animal-source foods. *Tubaramure* had a positive effect on maternal dietary diversity which was largely a result of the legumes in the program food rations. Among children, several complementary feeding practices were improved such as the proportion of children who consumed ≥4 food groups, received the minimum number of recommended meals, were fed a minimally acceptable diet, and consumed iron-rich foods.

*Tubaramure* increased household energy consumption by 622–681 kcal per AE/d. The program's food ration provided a beneficiary families with means of 495 (T18 arm) and 650 (T24 and TNFP arms) kcal per AE/d from birth to 24 mo (Supplemental Table 2). Because not all households participated in the program (impact estimates are intent-to-treat), the estimated impact on energy consumption among beneficiary families was larger than the net energy transfer provided by the program. The program's impact on fruit consumption, and the significant effect on legume and micronutrient consumption which remained when the ration foods were excluded, indicate that the program ration freed up some resources that were used to improve the quality of the household's regular diet. The food rations played a key role in *Tubaramure*’s impact on the diet of mothers and children as shown by our findings that the impact on maternal dietary diversity was smaller or disappeared when leaving out the program's CSB; the impact on the proportion of children consuming ≥4 food groups and iron-rich foods also disappeared when leaving out CSB; and the effect sizes on the proportion of children fed a minimally acceptable diet were considerably smaller without CSB. Lastly, as shown in our intrahousehold redistribution scenarios, *Tubaramure*’s impact on household consumption was large enough to potentially make a meaningful contribution to the micronutrient intake of mothers and young children.

The third study objective was to assess postprogram effects. *Tubaramure* had a postprogram effect across all treatment arms on household food security, maternal dietary diversity, and on the proportion of children consuming a minimally acceptable diet. Interestingly, the postprogram effect on maternal dietary diversity was driven by a substantial increase in non-vitamin A-rich fruit and vegetable intake. Other significant sustained effects including on household dietary diversity and on children consuming a minimally diverse diet and having minimal meal frequency were limited to select treatment arms. Households in the second follow-up survey (when the postprogram impact was assessed) had graduated from the program a mean of 8 mo before the survey; we assessed child feeding practices in children who had never been the targeted program beneficiary (younger siblings aged 0–24 mo of the children in our evaluation at endline). Thus, the positive results on children's complementary feeding practices could not have been because of the program's food transfers. Given the large increase in household energy consumption, it is unlikely that households were saving food rations or income from selling food rations for later consumption. These findings thus suggest a sustained effect of the program's BCC component and benefits on children beyond those that were direct program beneficiaries.

A limitation of our study is that some of the outcomes (e.g., household consumption) were not assessed at baseline, but it is unlikely that the estimated program impact was a result of pre-existing differences between study arms. Clusters were randomly assigned to the different study arms and the study arms appeared balanced on characteristics that were measured at baseline, many of which we expect to be strongly associated with the outcomes only assessed at follow-up. Another potential limitation is that individual dietary assessment was beyond the scope of this study. Per AE estimates based on household-level consumption data may mask inequitable intrahousehold allocation of foods and may be particularly inaccurate to estimate the dietary adequacy of young children (33, 34). Our simulations show, however, that the impact on household consumption had the potential to meaningfully improve the micronutrient intake of mothers and young children. Third, the high prevalence of hunger and food insecurity in this sample and the general thinness of the study population (mean maternal BMI was 20.7 kg/m^2^) indicate that many households in the study sample were energy-deficient (35). Thus, our estimate of mean household energy consumption in the control arm (around 3000 kcal per AE/d) may have slightly overestimated actual consumption. However, even if this was the case, this should not have biased the impact estimates which are based on differences between study arms. A final limitation is that the micronutrient estimates represent micronutrient availability in raw foods, that is they are not adjusted for micronutrient retention after preparation. This limits the interpretation of the results to raw micronutrient availability.

We are not aware of other studies that have rigorously assessed the impact of combined family and individual rations with BCC on household and individual consumption-related outcomes. This makes difficult comparing our findings to those of other studies. Evaluations of food aid programs have found that they increase household food consumption (5), but details on the magnitude of the impacts on energy consumption (relative to the caloric size of the transfer) or on consumption of specific food groups or nutrients are missing. Likewise, supplementary feeding trials have found positive impacts on anthropometric outcomes (7), but evidence on the impact of these interventions in a programmatic setting on diet-related outcomes is lacking. Our study starts to fill this evidence gap and provides important lessons for programs and research.

First, this program had a large impact on household energy consumption, but the effect on improving the quality of the diet was modest. No impact was found on energy from vegetables or animal-source foods, important sources of micronutrients and high-quality protein (in the case of animal-source foods). The program considerably increased the proportion of children aged 6–23.9 mo who consumed ≥4 food groups, but no (meaningful) impacts were found on their consumption of specific food groups rich in micronutrients or high-quality protein. The primary focus of households to increase staple food consumption is consistent with the high food insecurity in this population. Increasing the consumption of nutrient-rich foods may require a larger household transfer, a different type of transfer (e.g., in-kind transfers that include animal source foods or dairy products, or vouchers or cash if these products are available and affordable in markets), the inclusion of income-generating activities (which were not part of the *Tubaramure* program), and/or a stronger focus of the health benefits of these foods in the BCC strategy. A larger impact on the quality of the household and individual diet could have resulted in larger impacts on anemia, linear growth, and child development (9, 10, 36).

Second, the BCC appears to have contributed to the program's impact. Even though the integrated nature of the program does not allow us to fully separate the role of the BCC, the postprogram impacts suggest that BCC contributed to improvements in IYCF of the targeted child's younger sibling. Another recent evaluation of a multisectoral program in Burkina Faso documented the likely role that BCC played in increasing consumption (especially of micronutrients) (37). BCC exposure, however, was lower than intended by the program's design (10), and our process evaluation documented that quality was often suboptimal (the mothers who led the BCC sessions often missed adequate teaching skills and technical background to effectively carry out the BCC sessions). In addition, delays in the roll-out of the complementary feeding messages limited exposure to this important knowledge for a cohort of children (13). Thus, our findings suggest that even the limited exposure to BCC contributed to the program's impact and that a stronger BCC component could have led to even larger program impacts on IYCF practices.

Finally, the findings show that studying program impact on outcomes that are typically considered secondary in nature (38), but are part of the pathways of impact for maternal and child biological and developmental outcomes, provides important insights that would have been missed if we had limited our impact study to outcomes like anemia, linear growth retardation, and language and motor development (9, 10, 36).

In conclusion, *Tubaramure* had large and meaningful effects on improving household food security and increasing energy and micronutrient consumption in a severely resource-constrained population from rural Burundi. The program's effects on improving the diversity of household and maternal food consumption were limited, but the program improved several infant and young child complementary feeding practices. The sustained effects on household food security and maternal and child diets after the program ended confirm the importance of including effective BCC in food-assisted development programs.

## Supplementary Material

nxz295_Supplemental_File

## Notes

The study was made possible by the generous support of the American people through the support of the Office of Food for Peace, Bureau for Democracy, Conflict, and Humanitarian Assistance, and the Office of Health, Infectious Diseases, and Nutrition, Bureau for Global Health, U.S. Agency for International Development (USAID), under terms of Cooperative Agreement No. AID-OAA-A-12-00005, through the Food and Nutrition Technical Assistance III Project (FANTA), managed by FHI 360. This study also received support from the CGIAR Research Program on Agriculture for Nutrition and Health (A4NH), led by IFPRI.

JLL is a member of the editorial board of *Journal of Nutrition*.

Author disclosures: The authors report no conflicts of interest.

Supplemental Text and Supplemental Tables 1–7 are available from the “Supplementary data” link in the online posting of the article and from the same link in the online table of contents at https://academic.oup.com/jn.

Abbreviations used: AE, adult equivalent; BCC, behavior change communication; CSB, corn-soy blend; FA-MCHN, Food-Assisted Maternal and Child Health and Nutrition; FFP, USAID's Office of Food for Peace; HFIAS, household food insecurity access score; HHS, household hunger score; IYCF, infant and young child feeding; USAID, U.S. Agency for International Development.
