# Supplementary material for: Tubaramure, a Food-Assisted Maternal and Child Health and Nutrition Program in Burundi, Increased Household Food Security and Energy and Micronutrient Consumption, and Maternal and Child Dietary Diversity: A Cluster-Randomized Controlled Trial
Source: J Nutr. 2019 Dec 20;150(4):945–57. doi: 10.1093/jn/nxz295 (PMC7138675; doi:10.1093/jn/nxz295)
Supplement: nxz295_Supplemental_File [file nxz295_supplemental_file.docx]

**The impact of *Tubaramure*, a food-assisted maternal and child health and nutrition program in Burundi on household food security, consumption, and dietary diversity, and on maternal dietary diversity and child complementary feeding practices: a cluster-randomized controlled trial - Jef L Leroy et al. - Online Supplementary Material**

**Supplemental Text**^[[1]](#footnote-1)^

**The three program components of the *Tubaramure* program**

The core package of the *Tubaramure* program included three components: the distribution of food rations; improvements in the provision and use of health services; and a behavior change communication (BCC) strategy focused on improving health, hygiene and nutrition practices. The **food** component of the *Tubaramure* program aimed to increase household food security in terms of both quantity and quality (through a family ration containing micronutrient-fortified foods), and maternal and child nutrition (through the individual micronutrient-fortified foods ration targeted at pregnant and lactating mothers and children aged from 6 mo (up to 17.9 or 23.9 mo depending on the study group)). The monthly household food ration provided to all *Tubaramure* beneficiary households included 12 kg of corn–soy blend (CSB) and 1,200 g of vitamin A- and D-fortified vegetable oil (see **Supplemental Table 1** for micronutrient composition). An individual ration was provided in addition to the household food ration. For beneficiary mothers during pregnancy and during the first 6 mo of lactation, this ration included 6 kg of CSB and 600 g of oil; the child’s individual ration, which was provided starting at 6 mo – the age at which food should be introduced in the child’s diet in addition to breast milk – included 3 kg of CSB and 300 g of oil. Decisions about the types and quantities of food included in the rations were made by the consortium of NGOs.

The **health** component was designed to improve the provision of preventive health services by health staff and to increase the use of these services by pregnant and lactating women and children aged between 0 and 23.9 mo. The *Tubaramure* program trained health staff and provided some key supplies for implementing health services (e.g., equipment for prenatal care, labor and delivery, growth monitoring, and curative care). In addition, the program encouraged the use of preventive health services by pregnant and lactating women (i.e., pre and postnatal services, respectively) and children aged 0–23.9 mo (i.e., growth monitoring and promotion, vaccination) through the BCC strategy. Beneficiary mothers and children were expected to attend and use these recommended preventive health services at the local health center but attendance was not set as a condition for receipt of the program’s food assistance.

*Tubaramure*’s third component, the **BCC strategy**, was designed to encourage the adoption of best practices in health, hygiene, and nutrition. It was implemented by program staff, locally hired *Tubaramure* health promoters (THPs), and leader mothers who were selected as teachers by their fellow beneficiary mothers. The curriculum contained five modules, each with between 6 and 12 lessons. Lessons provided mothers with information on essential nutrition, hygiene and care practices during pregnancy (e.g., maternal nutrition and handwashing) and during infancy and young childhood (e.g., infant and young child feeding practices, danger signs of childhood illness and point-of-use water treatment), on the management of childhood illness (e.g., use of ORS and feeding of sick children), and on the importance of using bed nets. The *care groups* for leader mothers were held every 2 wk. During these group meetings, leader mothers were trained by the THP. Leader mothers in turn met with the beneficiary mothers in beneficiary mother care groups every 2 wk and discussed the topics they had most recently learned from the THP. Both types of care groups were supposed to consist of 10 to 12 mothers. Leader mothers used the program-designed illustrations to teach each BCC session and encouraged engagement from participants by asking open-ended questions. Other components of the BCC strategy included cooking demonstrations to show mothers how to prepare the CSB and how to cook more diverse meals and home visits to reinforce messages taught during the care group session.

As with the health services, beneficiary mothers were expected to attend the BCC sessions, but this was not enforced as a condition of receiving the food ration.

**Supplemental Table 1: Energy and micronutrient content per 100 g of corn-soy blend (CSB) and fortified vegetable oil**

|  | **CSB** | **Vegetable oil** |
| --- | --- | --- |
| Energy, kcal | 376 | 884 |
| Iron, mg | 17.49 | 0.05 |
| Zinc, mg | 5.00 | 0.01 |
| Vitamin A, μg RE | 784 | 1,800–2,250 |
| Folic acid, μg | 300 | - |
| Vitamin C, mg | 40 | - |
| Cu, mg | 0.90 | - |
| Calcium, mg | 831 | - |
| Phosphorous, mg | 206 | - |
| Potassium, mg | 634 | - |
| Magnesium, mg | 173.8 | - |
| Selenium, mg | 6 | - |
| Iodine, μg | 56.9 | - |
| Manganese, mg | 0.7 | - |
| Sodium, mg | 7.3 | - |
| Thiamin, mg | 0.53 | - |
| Riboflavin, mg | 0.48 | - |
| Niacin, mg | 6.23 | - |
| Pantothenic acid, mg | 3.4 | - |
| Vitamin B-6, mg | 0.5 | - |
| Vitamin B-12, μg | 1 | - |
| Vitamin D, IU | 198 | 1,700–2,100 |
| Vitamin E, mg | 8.70 | 8.18 |
| Vitamin K, μg | - | 183.9 |

**Supplemental Table 2: Calculation of the per adult equivalent energy provided by the *Tubaramure* food ration**

| **Beneficiary type in household** | **CSB per month** | | **Oil per month** | | **Total food ration** | | |
| --- | --- | --- | --- | --- | --- | --- | --- |
|  |  |  |  |  | **Per month** | **Per day** | **Per adult equivalent per day**^1^ |
|  | **Quantity**  **(kg)** | **Energy**  **(kcal)** | **Quantity**  **(kg)** | **Energy**  **(kcal)** | **Energy**  **(kcal)** | **Energy**  **(kcal)** | **Energy**  **(kcal)** |
| Beneficiary type |  |  |  |  |  |  |  |
| Pregnant mother | 18 | 67,680 | 1.8 | 15,912 | 83,592 | 2,748 | 743 |
| Mother of child under 6 mo | 18 | 67,680 | 1.8 | 15,912 | 83,592 | 2,748 | 743 |
| Child 6 to 23.9 mo | 15 | 56,400 | 1.5 | 13,260 | 69,660 | 2,290 | 619 |
| “Average” Tubaramure household^2^ |  |  |  |  |  |  |  |
| T24^3^ |  | 59,220 |  | 13,923 | 73,143 | 2,405 | 650 |
| T18 |  | 45,120 |  | 10,608 | 55,728 | 1,832 | 495 |
| TNFP |  | 59,220 |  | 13,923 | 73,143 | 2,405 | 650 |

^1^ The 2012 sample average of 3.7 adult equivalents per household was used.

^2^ Based on program participation data, we calculated a weighted sample average, using 6 months of participation during the first 6 mo of the child life and 18 mo from 6 to 23.9 mo in the T24 and TNFP arms and 12 months in T18.

^3^ The T24 arm received all program benefits during pregnancy and up to the age of 23.9 mo for the child. The same benefits were received in the T18 arm, but food rations were discontinued when the child was 17.9 mo of age. The TNFP (“no food during pregnancy”) arm started receiving food rations at birth, but the other benefits were the same as in T24.

**Supplemental Table 3: Impact of *Tubaramure* on household daily energy and micronutrient consumption per adult equivalent (excluding CSB and *Tubaramure* oil consumption)**

|  | **Follow-up (2012)** | | | | **Impact^2^** | | |
| --- | --- | --- | --- | --- | --- | --- | --- |
| **Table 2 paper** | **Study arm^1^** | | | |  | | |
|  | **T24**^3^ | **T18** | **TNFP** | **Control** | **T24 vs control** | **T18 vs control** | **TNFP vs control** |
| *n* | 389 | 804 | 391 | 778 | 2362 | 2362 | 2362 |
| Ln energy consumed, ln(kcal) | 7.9±0.6 | 8.0±0.6 | 7.9±0.5 | 7.9±0.5 | 0.0±0.1 | 0.1±0.1* | 0.1±0.1 |
| Total energy consumed, kcal | 3181.7±1915.9 | 3307.8±1819.1 | 3155.3±1688.1 | 2979.8±1628.9 | 201.9±288.1 | 328.0±194.7** | 175.5±192.4 |
| Energy from |  |  |  |  |  |  |  |
| cereals and grains, kcal | 306.3±435.5 | 351.3±442.6 | 371.1±510.4 | 314.0±450.3 | -7.7±48.5 | 37.3±34.4 | 57.1±51.7 |
| roots and tubers, kcal | 1776±1,464 | 1831±1368 | 1664±1150 | 1803±1302 | -27.2±189.2 | 28.0±155.5 | -139.3±146.7 |
| legumes, nuts and pulses | 521.5±408.0 | 542.8±470.5 | 546.0±457.7 | 418.6±357.4 | 102.9±60.1* | 124.1±48.1** | 127.3±51.3** |
| fruits, kcal | 205.0±452.4 | 188.7±300.6 | 191.9±339.3 | 116.3±196.4 | 88.7±33.1** | 72.4±24.8** | 75.7±30.9** |
| vegetables, kcal | 125.7±194.5 | 139.2±269.1 | 120.1±212.5 | 111.1±179.0 | 14.6±16.4 | 28.0±22.0 | 8.9±20.2 |
| meat and poultry, kcal | 16.2±40.4 | 19.3±50.3 | 20.8±57.8 | 18.6±58.8 | -2.4±3.8 | 0.7±3.0 | 2.2±4.5 |
| fish and seafood, kcal | 13.0±36.8 | 14.5±35.6 | 9.6±23.8 | 12.4±30.4 | 0.6±3.9 | 2.1±2.0 | -2.8±2.1 |
| milk and dairy, kcal | 4.5±26.4 | 5.3±29.5 | 7.2±37.0 | 3.8±26.4 | 0.8±1.7 | 1.5±1.6 | 3.4±2.3 |
| eggs, kcal | 1.4±14.8 | 0.6±3.3 | 0.6±2.5 | 0.3±1.8 | 1.1±0.8 | 0.3±0.2 | 0.2±0.2 |
| oils and fats, kcal | 144.3±145.9 | 156.0±274.7 | 158.0±210.3 | 144.2±305.6 | 0.0±15.0 | 11.8±13.4 | 13.8±14.1 |
| sugars, kcal | 19.5±81.3 | 15.4±55.5 | 17.4±73.0 | 6.0±28.8 | 13.5±5.6** | 9.5±3.9** | 11.4±4.8** |
| miscellaneous, kcal | 48.5±74.4 | 43.6±82.0 | 48.9±79.3 | 31.4±67.1 | 17.1±7.2** | 12.2±5.3** | 17.5±6.4** |
| Micronutrients^4^ |  |  |  |  |  |  |  |
| Vitamin C, mg | 210±137 | 231.0±139 | 227±134 | 205±124 | 4.9±13.5 | 25.8±18.3* | 22.1±16.9* |
| Iron, mg | 21.9±12.0 | 23.2±13.0 | 22.1±12.1 | 20.8±11.9 | 1.2±1.7 | 2.4±1.3** | 1.3±1.3 |
| Vitamin A, μg | 1390±1520 | 1190±1290 | 1230±1380 | 1090±1520 | 299±201* | 98.7±130 | 136±187 |
| Zinc, mg | 10.6±5.9 | 10.9±6.0 | 10.6±5.8 | 9.6±5.1 | 0.9±0.8 | 1.3±0.5** | 1.0±0.6** |
| Thiamin, mg | 2.5±1.3 | 2.6±1.4 | 2.6±1.4 | 2.3±1.2 | 0.3±0.2* | 0.4±0.1** | 0.3±0.1** |
| Riboflavin, mg | 1.8±1.1 | 1.7±1.0 | 2.6±1.4 | 1.5±0.9 | 0.2±0.1** | 0.2±0.1** | 0.2±0.1* |
| Vitamin B6, mg | 4.3±2.9 | 4.2±2.4 | 4.3±2.5 | 3.7±2.2 | 0.6±0.3** | 0.5±0.2** | 0.6±0.2** |
| Folate, μg | 1010±556 | 1050±613 | 1030±590 | 879±500 | 134±95.6* | 173±73.7** | 156±71.1** |
| Vitamin B12, μg | 0.4±0.7 | 0.4±0.7 | 0.4±0.8 | 0.4±0.8 | 0.0±0.1 | 0.0±0.0 | 0.0±0.1 |

^1^ Values are mean ± SD or %.

^2^ Values are single difference impact estimates ± SE. All estimates controlled for clustering, household head's education and occupation, maternal education, household size and housing ownership. One-sided tests were conducted when there was an *a priori* hypothesis about the direction of program effect.

^3^ The T24 arm received all program benefits during pregnancy and up to the age of 23.9 mo for the child. The same benefits were received in the T18 arm, but food rations were discontinued when the child was 17.9 mo of age. The TNFP (“no food during pregnancy”) arm started receiving food rations at birth, but the other benefits were the same as in T24.

^4^ Values represent micronutrient availability in raw foods, unadjusted for micronutrient retention after preparation.

* Impact estimate significantly different from 0, p<0.10.

** Impact estimate significantly different from 0, p<0.05.

**Supplemental Table 4: Simulated impact of *Tubaramure* on mothers’ daily micronutrient consumption under three intrahousehold distribution scenarios**

|  | **Requirements**  **(19 - 50 y old lactating woman)**^1^ | **Share proportional to adult equivalent**^2,3^ | | | **Share proportional to 1.5 times adult equivalent**^2,3^ | | | **Share proportional to 0.5 times adult equivalent**^2,3^ | | |
| --- | --- | --- | --- | --- | --- | --- | --- | --- | --- | --- |
|  |  | **T24^4^ vs control** | **T18 vs control** | **TNFP vs control** | **T24 vs control** | **T18 vs control** | **T24 vs control** | **T18 vs control** | **TNFP vs control** | **TNFP vs control** |
| Vitamin C, mg | 120 | 38.2±13.4** | 48.6±16.2** | 51.6±16.1** | 76.4±26.8** | 97.2±32.5** | 103.3±32.1** | 19.1±6.7** | 24.3±8.1** | 25.8±8.0** |
| Iron, mg | 9 | 15.6±2.7** | 12.6±1.3** | 13.7±1.7** | 31.3±5.4** | 25.3±2.7** | 27.5±3.3** | 7.8±1.4** | 6.3±0.7** | 6.9±0.8** |
| Vitamin A, μg | 1300 | 933±191** | 574±127** | 709±185** | 1870±381** | 1150±254** | 1420±370** | 467±95.3** | 287±63.4** | 355±92.5** |
| Zinc, mg | 10 | 5.0±1.1** | 4.2±0.6** | 4.5±0.6** | 10.1±2.1** | 8.4±1.1** | 9.0±1.2** | 2.5±0.5** | 2.1±0.3** | 2.2±0.3** |
| Thiamin, mg | 1.4 | 0.7±0.2** | 0.6±0.1** | 0.7±0.1** | 1.4±0.4** | 1.3±0.2** | 1.4±0.2** | 0.3±0.1** | 0.3±0.1** | 0.3±0.1** |
| Riboflavin, mg | 1.6 | 0.6±0.1** | 0.5±0.1** | 0.5±0.1** | 1.3±0.3** | 1.0±0.2** | 1.0±0.2** | 0.3±0.1** | 0.2±0.0** | 0.3±0.1** |
| Vitamin B6, mg | 2.0 | 1.0±0.3** | 0.8±0.2** | 0.9±0.2** | 2.0±0.6** | 1.6±0.4** | 1.9±0.4** | 0.5±0.2** | 0.4±0.1** | 0.5±0.1** |
| Folate, μg | 500 | 371±103** | 337±67.9** | 356±66.6** | 742±206.3** | 675±136** | 712±133** | 186±51.6** | 169±33.9** | 178±33.3** |
| Vitamin B12, μg | 2.8 | 0.8±0.1* | 0.6±0.1* | 0.7±0.1* | 1.7±0.3* | 1.3±0.1* | 1.5±0.2* | 0.4±0.1* | 0.3±0.0* | 0.4±0.0* |

^1^ Recommended dietary allowances (RDA) are from the Institute of Medicine (1) except zinc, which was taken from International Zinc Nutrition Consultative Group (2).

^2^ Values are single difference impact estimates ± SE. All estimates controlled for clustering, household head's education and occupation, maternal education, household size and housing ownership. One-sided tests were conducted when there was an *a priori* hypothesis about the direction of program effect.

^3^ Values represent micronutrient availability in raw foods, unadjusted for micronutrient retention after preparation.

^4^ The T24 arm received all program benefits during pregnancy and up to the age of 23.9 mo for the child. The same benefits were received in the T18 arm, but food rations were discontinued when the child was 17.9 mo of age. The TNFP (“no food during pregnancy”) arm started receiving food rations at birth, but the other benefits were the same as in T24.

* Impact estimate significantly different from 0, p<0.10.

** Impact estimate significantly different from 0, p<0.05.

**Supplemental Table 5: Simulated impact of *Tubaramure* on childrens’ daily micronutrient consumption under three intrahousehold distribution scenarios**

|  | **Requirements** ^1^  **(6 to 11.9 mo old child)** | | **Share proportional to adult equivalent**^2,3^ | | | **Share proportional to 1.5 times adult equivalent**^2,3^ | | | **Share proportional to 0.5 times adult equivalent**^2,3^ | | |
| --- | --- | --- | --- | --- | --- | --- | --- | --- | --- | --- | --- |
|  | **Breastfed** | **Non- breastfed** | **T24^4^ vs control** | **T18 vs control** | **TNFP vs control** | **T24 vs control** | **T18 vs control** | **TNFP vs control** | **T24 vs control** | **T18 vs control** | **TNFP vs control** |
| Vitamin C, mg | 50 | 0 | 10.5±3.9** | 12.9±4.3** | 11.9±3.7** | 21.1±7.7** | 25.7±8.6** | 23.8±7.5** | 5.3±1.9** | 6.4±2.2** | 6.0±1.9** |
| Iron, mg | 11 | 20.8 | 4.4±0.8** | 3.4±0.4** | 3.7±0.5** | 8.8±1.6** | 6.8±0.7** | 7.4±1.0** | 2.2±0.4** | 1.7±0.2** | 1.9±0.3** |
| Vitamin A, μg | 500 | 3 | 256±56.3** | 145±38.3** | 179±58.6** | 512±112.6** | 294±76.6** | 359±117.1** | 128±28.2** | 73.4±19.2** | 89.7±29.3** |
| Zinc, mg | 4.0 | 2.3 | 1.4±0.3** | 1.1±0.1** | 1.2±0.2** | 2.8±0.6** | 2.3±0.3** | 2.4±0.4** | 0.7±0.2** | 0.6±0.1** | 0.6±0.1** |
| Thiamin, mg | 0.3 | 0.15 | 0.2±0.1** | 0.2±0.0** | 0.2±0.0** | 0.4±0.1** | 0.3±0.1** | 0.3±0.1** | 0.1±0.0** | 0.1±0.0** | 0.1±0.0** |
| Riboflavin, mg | 0.4 | 0.2 | 0.2±0.0** | 0.1±0.0** | 0.1±0.0** | 0.3±0.1** | 0.3±0.0** | 0.3±0.1** | 0.1±0.0** | 0.1±0.0** | 0.1±0.0** |
| Vitamin B6, mg | 0.3 | 0 | 0.4±0.1** | 0.2±0.1** | 0.2±0.1** | 0.5±0.2** | 0.4±0.1** | 0.4±0.1** | 0.1±0.0** | 0.1±0.0** | 0.1±0.0** |
| Folate, μg | 80 | 0 | 104±30.0** | 92.2±17.6** | 95.7±19.6** | 209±60.0** | 184±35.1** | 191±39.3** | 52.2±15.0** | 46.1±8.8** | 47.8±9.8** |
| Vitamin B12, μg | 0.5 | 0 | 0.2±0.0* | 0.2±0.0* | 0.2±0.0* | 0.5±0.1* | 0.3±0.0* | 0.4±0.1* | 0.1±0.0* | 0.1±0.0* | 0.1±0.0* |

^1^ Recommended dietary allowances (RDA) are from the Institute of Medicine (1) except zinc, which was taken from International Zinc Nutrition Consultative Group (2). No RDA exist for infants for vitamin C and folate. The requirements were set as the Adequate Intake (AI) from the Institute of Medicine (1). For breastfed infants, nutrient requirements represent those obtained from complementary food only; average values for 6-8 and 9-11 months from Table 26 from WHO (1998) were used (3).

^2^ Values are single difference impact estimates ± SE. All estimates controlled for clustering, household head's education and occupation, maternal education, household size and housing ownership. One-sided tests were conducted when there was an *a priori* hypothesis about the direction of program effect.

^3^ Values represent micronutrient availability in raw foods, unadjusted for micronutrient retention after preparation.

^4^ The T24 arm received all program benefits during pregnancy and up to the age of 23.9 mo for the child. The same benefits were received in the T18 arm, but food rations were discontinued when the child was 17.9 mo of age. The TNFP (“no food during pregnancy”) arm started receiving food rations at birth, but the other benefits were the same as in T24.

* Impact estimate significantly different from 0, p<0.10.

** Impact estimate significantly different from 0, p<0.05.

**Supplemental table 6: Post-program impact of *Tubaramure* on household food insecurity, hunger, and dietary diversity**

|  | **Baseline (2010)** | | | | **Follow-up (2014)** | | | | **Impact^2^** | | |
| --- | --- | --- | --- | --- | --- | --- | --- | --- | --- | --- | --- |
| **Table 2 paper** | **Study arm^1^** | | | | **Study arm^1^** | | | |  | | |
|  | **T24^3^** | **T18** | **TNFP** | **Control** | **T24** | **T18** | **TNFP** | **Control** | **T24 vs control** | **T18 vs control** | **TNFP vs control** |
| *n* |  |  |  |  | 946 | 955 | 957 | 550 | 3408 | 3408 | 3408 |
| HFIAS Score |  |  |  |  | 10.6±7.4 | 11.0±7.1 | 10.5±7.0 | 13.2±7.2 | -2.6±1.0** | -2.2±0.9** | -2.7±0.9** |
| HFIAS Scale: Food secure |  |  |  |  | 13.3 | 11.4 | 12.7 | 7.3 | 6.0±1.9** | 4.1±1.8** | 5.5±2.3** |
| *n* | 979 | 580 | 980 | 988 | 949 | 961 | 958 | 551 | 6946 | 6946 | 6946 |
| HHS Score | 1.2±1.5 | 1.5±1.5 | 1.4±1.5 | 1.6±1.5 | 0.7±1.3 | 0.8±1.3 | 0.6±1.3 | 1.1±1.6 | 0.0±0.2 | -0.1±0.2 | -0.2±0.2* |
| HHS Scale: Little-no hunger | 59.9 | 54.3 | 53.7 | 48.6 | 79.9 | 78.4 | 80.9 | 70.8 | -1.9±4.2 | 0.2±4.3 | 3.5±4.4 |
| Diet diversity score | 4.1±1.7 | 4.1±1.6 | 4.1±1.7 | 3.8±1.6 | 4.7±1.6 | 4.9±1.6 | 5.1±1.6 | 4.3±1.5 | 0.2±0.2 | 0.3±0.2* | 0.6±0.2** |

^1^ Values are mean ± SD or %.

^2^ Values are double difference impact estimates ± SE for outcomes with baseline and follow-up data, and single difference impact estimates ± SE for outcomes with follow-up data only. All estimates controlled for clustering, household head's education and occupation, maternal education, household size and housing ownership. One-sided tests were conducted when there was an *a priori* hypothesis about the direction of program effect.

^3^ The T24 arm received all program benefits during pregnancy and up to the age of 23.9 mo for the child. The same benefits were received in the T18 arm, but food rations were discontinued when the child was 17.9 mo of age. The TNFP (“no food during pregnancy”) arm started receiving food rations at birth, but the other benefits were the same as in T24.

* Impact estimate significantly different from 0, p<0.10.

** Impact estimate significantly different from 0, p<0.05.

**Supplemental Table 7: Post-program impact of *Tubaramure* on mothers’ and children’s dietary diversity and child feeding practices**

|  | **Baseline (2010)** | | | | **Follow-up (2014)** | | | | **Impact^2^** | | |
| --- | --- | --- | --- | --- | --- | --- | --- | --- | --- | --- | --- |
| **Table 2 paper** | **Study arm^1^** | | | | **Study arm^1^** | | | |  | | |
|  | **T24^3^** | **T18** | **TNFP** | **Control** | **T24** | **T18** | **TNFP** | **Control** | **T24 vs control** | **T18 vs control** | **TNFP vs control** |
| *Mothers* |  |  |  |  |  |  |  |  |  |  |  |
| *n* |  |  |  |  | 920 | 941 | 943 | 542 | 3346 | 3346 | 3346 |
| Dietary diversity score |  |  |  |  | 4.6±1.1 | 4.6±1.2 | 4.7±1.1 | 4.2±1.2 | 0.4±0.1** | 0.4±0.1** | 0.5±0.1** |
| *% of mothers who consumed (past 24 h)* |  |  |  |  |  |  |  |  |  |  |  |
| *n* |  |  |  |  | 921 | 942 | 948 | 545 | 3356 | 3356 | 3356 |
| Starchy staples |  |  |  |  | 93.5 | 94.3 | 95.6 | 93.0 | 0.5±3.2 | 1.2±3.2 | 2.5±3.0 |
| *n* |  |  |  |  | 921 | 943 | 947 | 546 | 3357 | 3357 | 3357 |
| Legumes, nuts and seeds |  |  |  |  | 79.4 | 78.3 | 84.9 | 72.2 | 7.2±5.1 | 6.1±4.6 | 12.7±4.9** |
| *n* |  |  |  |  | 921 | 943 | 948 | 546 | 3358 | 3358 | 3358 |
| Dark green leafy vegetables |  |  |  |  | 87.9 | 91.9 | 89.3 | 83.2 | 4.8±3.2 | 8.8±2.8** | 6.2±3.4* |
| *n* |  |  |  |  | 921 | 943 | 947 | 546 | 3357 | 3357 | 3357 |
| Other vitamin A-rich fruits and vegetables |  |  |  |  | 87.7 | 89.2 | 90.0 | 88.8 | -1.1±3.1 | 0.4±3.0 | 1.1±2.7 |
| *n* |  |  |  |  | 921 | 943 | 947 | 545 | 3356 | 3356 | 3356 |
| Other fruits and vegetables |  |  |  |  | 76.8 | 68.0 | 71.2 | 55.4 | 21.4±5.7** | 12.6±6.5* | 15.8±5.7** |
| *n* |  |  |  |  | 921 | 943 | 945 | 546 | 3355 | 3355 | 3355 |
| Organ meat |  |  |  |  | 1.2 | 0.7 | 1.4 | 0.4 | 0.8±0.7 | 0.4±0.5 | 1.0±0.6* |
| *n* |  |  |  |  | 920 | 942 | 946 | 546 | 3354 | 3354 | 3354 |
| Meat and fish |  |  |  |  | 30.3 | 36.2 | 34.6 | 29.1 | 1.2±5.8 | 7.1±6.4 | 5.4±7.4 |
| *n* |  |  |  |  | 921 | 942 | 947 | 544 | 3354 | 3354 | 3354 |
| Eggs |  |  |  |  | 2.1 | 1.4 | 1.7 | 0.6 | 1.5±0.7** | 0.8±0.6 | 1.1±0.6* |
| *n* |  |  |  |  | 921 | 943 | 947 | 546 | 3357 | 3357 | 3357 |
| Milk and dairy products |  |  |  |  | 1.6 | 3.3 | 3.4 | 1.3 | 0.3±0.7 | 2.0±0.9** | 2.1±1.5 |
| *Children* |  |  |  |  |  |  |  |  |  |  |  |
| *n* | 315 | 677 | 320 | 672 | 173 | 188 | 184 | 79 | 2608 | 2608 | 2608 |
| Dietary diversity score | 2.6±1.2 | 2.6±1.3 | 2.7±1.3 | 2.4±1.2 | 3.0±1.5 | 2.8±1.6 | 2.9±1.5 | 2.6±1.5 | 0.2±0.2 | 0.1±0.2 | 0.0±0.2 |
| *% of children who consumed (past 24 h)* |  |  |  |  |  |  |  |  |  |  |  |
| *n* | 318 | 682 | 327 | 680 | 173 | 188 | 185 | 80 | 2634 | 2634 | 2634 |
| Grains, roots and tubers | 67.9 | 71.7 | 67.3 | 68.4 | 71.7 | 68.3 | 69.2 | 70.0 | -1.7±5.4 | -7.1±5.9 | -2.0±6.3 |
| *n* | 318 | 683 | 326 | 682 | 173 | 188 | 185 | 80 | 2635 | 2635 | 2635 |
| Legumes and nuts | 53.1 | 41.1 | 49.7 | 34.5 | 56.1 | 57.4 | 55.7 | 45.0 | -6.3±6.4 | 2.5±8.2 | -6.9±7.5 |
| *n* | 316 | 681 | 324 | 679 | 173 | 188 | 185 | 80 | 2626 | 2626 | 2626 |
| Milk and dairy products | 2.5 | 3.2 | 2.8 | 1.5 | 2.3 | 4.8 | 2.7 | 5.0 | -3.4±2.4 | -2.4±2.5 | -2.8±2.3 |
| *n* | 317 | 684 | 321 | 681 | 173 | 188 | 184 | 80 | 2628 | 2628 | 2628 |
| Flesh foods | 11.0 | 12.9 | 15.9 | 12.9 | 20.2 | 20.7 | 21.2 | 16.3 | 5.7±7.8 | 4.5±7.7 | 2.2±7.9 |
| *n* | 318 | 683 | 326 | 682 | 173 | 188 | 185 | 80 | 2635 | 2635 | 2635 |
| Eggs | 0.9 | 1.6 | 1.8 | 0.9 | 2.3 | 2.1 | 1.1 | 1.3 | 1.7±1.5 | 0.6±1.7 | -0.6±1.6 |
| *n* | 318 | 684 | 326 | 682 | 173 | 188 | 185 | 79 | 2635 | 2635 | 2635 |
| Vitamin A-rich fruits and vegetables | 85.8 | 88.5 | 87.7 | 88.0 | 82.1 | 76.1 | 80.0 | 79.7 | 1.9±5.5 | -5.1±5.2 | -2.6±6.1 |
| *n* | 318 | 683 | 326 | 681 | 173 | 189 | 185 | 80 | 2635 | 2635 | 2635 |
| Other fruits and vegetables | 41.2 | 36.5 | 41.4 | 37.3 | 64.7 | 55.0 | 60.5 | 45.0 | 17.1±6.8** | 12.7±8.6 | 11.9±5.9** |
| *Infant and young child feeding practices* |  |  |  |  |  |  |  |  |  |  |  |
| *% of children with* |  |  |  |  |  |  |  |  |  |  |  |
| *n* | 320 | 687 | 333 | 689 | 171 | 187 | 182 | 80 | 2649 | 2649 | 2649 |
| minimum dietary diversity (>=4) | 25.3 | 22.7 | 28.5 | 19.6 | 45.0 | 43.3 | 43.4 | 28.7 | 10.8±6.1** | 9.4±8.1 | 4.3±6.9 |
| *n* | 290 | 636 | 291 | 642 | 154 | 168 | 170 | 72 | 2423 | 2423 | 2423 |
| minimum meal frequency | 37.6 | 33.8 | 24.4 | 32.7 | 63.0 | 56.5 | 63.5 | 51.4 | 12.6±9.4* | 6.7±9.0 | 24.1±9.4** |
| *n* | 314 | 681 | 315 | 678 | 173 | 189 | 183 | 79 | 2612 | 2612 | 2612 |
| minimum acceptable diet | 11.1 | 7.5 | 7.6 | 6.8 | 30.1 | 29.6 | 27.3 | 13.9 | 13.8±4.7** | 14.6±5.7** | 13.7±6.4** |
| *n* | 317 | 683 | 321 | 681 | 172 | 188 | 184 | 80 | 2626 | 2626 | 2626 |
| iron-rich foods consumption | 12.9 | 13.0 | 16.8 | 13.1 | 20.9 | 22.9 | 26.6 | 17.5 | 3.0±8.5 | 5.7±8.0 | 6.3±9.1 |

^1^ Values are mean ± SD or %.

^2^ Values are double difference impact estimates ± SE for outcomes with baseline and follow-up data, and single difference impact estimates ± SE for outcomes with follow-up data only. All estimates controlled for clustering, household head's education and occupation, maternal education, household size and housing ownership. One-sided tests were conducted when there was an *a priori* hypothesis about the direction of program effect.

^3^ The T24 arm received all program benefits during pregnancy and up to the age of 23.9 mo for the child. The same benefits were received in the T18 arm, but food rations were discontinued when the child was 17.9 mo of age. The TNFP (“no food during pregnancy”) arm started receiving food rations at birth, but the other benefits were the same as in T24.

* Impact estimate significantly different from 0, p<0.10.

** Impact estimate significantly different from 0, p<0.05.

1. Abbreviations used: BCC: behavior change communication; CSB: corn-soy blend; HAZ: height-for-age Z-score; NGO: non-governmental organization; THP: *Tubaramure* health promotor; [↑](#footnote-ref-1)
